# Comparing the Trends of Vector-Borne Diseases (VBDs) before and after the COVID-19 Pandemic and Their Spatial Distribution in Southern Iran

**DOI:** 10.1155/2023/7697421

**Published:** 2023-09-15

**Authors:** Zahra Gheibi, Mitra Boroomand, Aboozar Soltani

**Affiliations:** ^1^Department of Epidemiology, School of Health, Shiraz University of Medical Sciences, Shiraz, Iran; ^2^Department of Medical Entomology and Vector Control, School of Health, Shiraz University of Medical Sciences, Shiraz, Iran; ^3^Research Center for Health Sciences, Institute of Health, Department of Medical Entomology and Vector Control, School of Health, Shiraz University of Medical Sciences, Shiraz, Iran

## Abstract

**Objectives:**

We aimed to model and predict the changes in the trend of vector-borne diseases (VBDs) before and after the COVID-19 pandemic in a high-risk area of Iran.

**Methods:**

This case-series study was conducted in Fars province, south of Iran, between April 2016 and July 2021. All referred cases of VBDs were considered during the five years to investigate the effect of the lockdown on the epidemiological profile of these diseases. We used time-series autoregressive integrated moving average (ARIMA) and seasonal ARIMA (SARIMA) models.

**Results:**

Pediculosis incidence trend was rising with a peak of 1,146 per 100,000 in 2018, followed by a dramatic decrease reached to the minimum amount of 157.8 per 100,000 in 2021. In contrast, malaria and scabies had a smooth decreasing trend ranging from 2.2 per 100,000 and 7.3 per 100,000 in 2016 to a minimum of 0.2 per 100,000 in 2021, respectively. Likewise, leishmaniasis had a falling trend, with a maximum rate of 82.9 per 100,000 in 2016 to the lowest rate of 9.4 per 100,000 in 2021. However, the difference between observed and expected values revealed that the consequences of the COVID-19 pandemic had increased the number of leishmaniasis cases.

**Conclusion:**

Tropical regions of Iran, including Fars province, are the favorite destinations for travelers. During COVID-19 outbreaks, some reasons, such as quarantine, movement restrictions, and social distancing, reduced human-vector contact and finally led to the reduction of VBDs in this area.

## 1. Introduction

Vector-borne diseases (VBDs) are health issues caused by disease agents and parasites in human populations that Arthropoda transmits. VBDs infect more than one billion people annually. Moreover, more than one million people die from these important diseases including malaria, dengue, trypanosomiasis, leishmaniasis, chagas disease, yellow fever, and other VBDs especially in tropical areas [1].

Factors such as climate change, global warming, the growing trend of international trade, traveling, the overuse of pesticides, the resistance of vectors to common insecticides, and the human population affect the prevalence of vector-borne diseases [2, 3]. In late 2019, pneumonia cases of unknown cause were reported in the Wuhan (China), and epidemiological surveys showed they were linked to the Wuhan Seafoods and live animals vendors market [4]. In less than five months, COVID-19 (coronavirus disease 2019) has grown from a limited area in Wuhan to more than 5 million people in almost all countries [5].

Some efforts have been made to control this unknown and invasive disease, such as quarantine limitations, which have affected human contacts and the environment. Therefore, it is expected that reports of vector-borne infections would change during the pandemic. A study was performed in Malaysia to determine the impact of COVID-19 lockdown on dengue transmission. The results showed the incidence trend of this VBD in the first two weeks of restrictions (the first stage of quarantine) was significantly reduced, while in the second stage, the incidence of dengue increased exponentially [6].

In Iran, however, the prevalence of VBDs is different because of the climatic diversity and various geographical regions. According to recent research, malaria transmission still exists in some areas of southern and southeastern Iran, especially in spring and autumn [7]. Malaria parasites are microorganisms that belong to the genus *Plasmodium* that it is transmitted through the bite of some female *Anopheles* mosquito [8].

There were estimated 241 million malaria cases worldwide in 85 malaria-endemic countries in 2020 [9]. Leishmaniasis is a VBD affecting the populations of high-risk areas in over 90 countries throughout Asia, Africa, the Middle East, and Central and South America. *Leishmania* parasites are transmitted through the bite of *phlebotomine* sand flies [10]. In recent decades, the prevalence of leishmaniasis has been rising in Iran because of drastic changes in some demographic factors and climate conditions [11].

According to previous studies, the scabies infestation rate ranges between 1.3% and 57% [12]. *Sarcoptes scabiei*, also called itch mite, is an ectoparasite that burrows canals into the skin and causes scabies. Pruritus is a common symptom of this parasitic disease resulting from an allergic hypersensitivity reaction to the mites, eggs, and fecal pellets [13, 14]. Lice are blood-feeder parasites that infest the body and head of human beings (pediculosis), especially in people with poor health conditions. In addition, school students, especially girls, are another susceptible population to the disease. Overall, 52,342 students (from primary and secondary schools) and around 20,000 adult persons were infested with *Pediculus capitis* in Iran for three decades (1990–2020) [15].

Recent studies showed that the world has changed since the beginning of the COVID-19 pandemic, and human interventions have accelerated these changes. Therefore, we expect to see dramatic changes in the pattern of vector-borne diseases, but there is no study has been carried out in this regard. In the present study, we aimed to model and predict the amount of change in the trend of main vector-borne diseases (leishmaniasis, pediculosis, malaria, and scabies) before and after the occurrence of the COVID-19 pandemic. Then, we decided to address this phenomenon in a critical high-risk area for VBDs in Iran.

## 2. Materials and Methods

This case-series study was conducted in Fars province between April 2016 and July 2021. Fars province, with an area of 122,400 km^2^, is located in the southwest of Iran with geographical locations 29°37′30″N, 52°31′54″E. Information on main reported cases of vector-borne disease was collected from the data registry designed by Iran's Ministry of Health and Medical Education. During these years, 153,550 cases of pediculosis, 10,686 cases of leishmaniasis, 296 cases of malaria, and 820 cases of scabies were reported in the studied area. The study region was all cities of Fars province except Fasa, Jahrom, Lar, and Gerash (these cities are not covered by Shiraz University of Medical Sciences and have a separate registry system).

### 2.1. Data Analysis

All referred cases of the main VBDs were considered during the five years (before and after the COVID-19 pandemic) to investigate the effect of lockdown on the frequency of these diseases. We used time-series autoregressive integrated moving average (ARIMA (*p*, *d*, *q*)) and seasonal ARIMA (SARIMA (*p*, *d*, *q*)(*P*, *D*, *Q*) *s*) models, where“AR (*p*)” represents the number ofautoregressive terms, “*I* (*d*)” is the difference in the nonseasonal observations, and “MA (*q*)” shows the size of the moving average. Also, the “*P*,” “*D*,” and “*Q*” parameters are similar to the “*p*,” “*d*,” and “*q*” parameters, but for the seasonal component of the data, the “*s*” parameter represents the length of the seasonal cycle. The monthly number of reported cases before the pandemic (from April 2016 to January 2020) was modeled to predict how the trend was if there was no COVID-19 pandemic (expected values). Then, the observed and expected values of 2020 and 2021 (during the COVID-19 pandemic) were compared with independent sample *T*-tests to find the effect of lockdown. To assess the time-series model, ITSM (interactive time series modeling) software (v. 2000) was used. This software is interactive Windows-based menu-driven software for time-series modeling and forecasting [16]. To assess the goodness of fit in SARIMA models, we chose the model with minimum Akaike information criterion (AIC), and after fitting the model, we checked the normality of residuals. All models were fitted appropriately, and residuals were normal. Also, after predicting the trend of each vector-borne disease, we considered the Arc Gis software to map the 5-year cumulative incidence rate of infections to find the high-risk regions in Fars province. The high-risk regions were determined using geometrical interval method. This algorithm creates geometric intervals by minimizing the sum of squares of the number of elements in each class. For calculating incidence rate, the denominators (people at risk) were estimated based on Iranian census in the site of statistical center of Iran. The confidence level for all statistical tests was considered as 0.95% in this study.

## 3. Results

The annual incidence of vector-borne diseases is reported in Table 1. As can be seen, the incidence of pediculosis (head lice) in the population increased over time, peaking in 2018 with 1,146 cases per 100,000 people. After 2018, there was a sharp decline in pediculosis, dropping to just 157.8 cases per 100,000 in 2021. In contrast, malaria and scabies infections showed a gradual yet consistent decreasing trend between 2016 and 2021. Malaria dropped from 2.2 cases to 0.2 cases per 100,000, and scabies declined from 7.3 to a minimum of 0.2 cases per 100,000 during the 2016–2021 periods. Similarly, the incidence of leishmaniasis infections exhibited an overall downward trend, reaching a high of 82.9 cases per 100,000 people in 2016 and then decreasing to a low of 9.4 cases per 100,000 by 2021.

**Figure 1:**
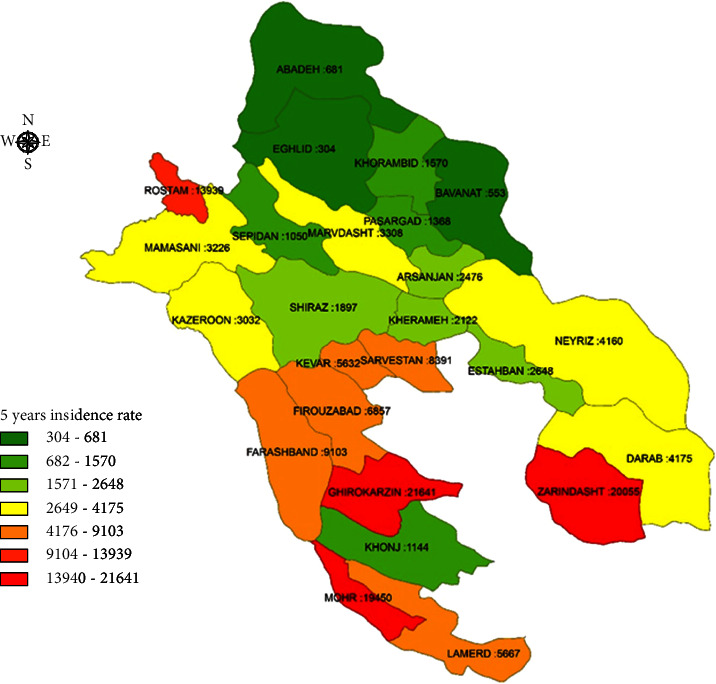
5-year incidence rate of pediculusis in Fars province.

**Figure 2:**
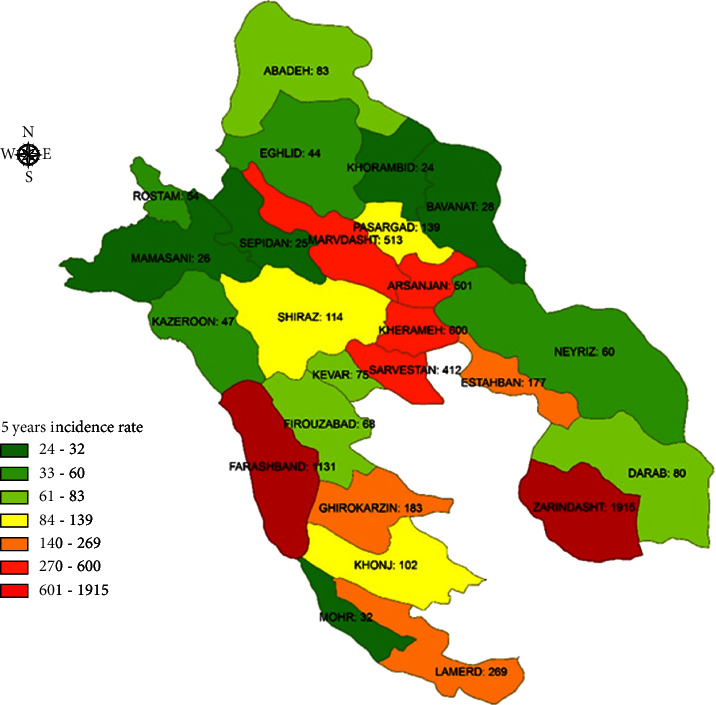
5-year incidence rate of leishmaniasis in Fars province.

**Figure 3:**
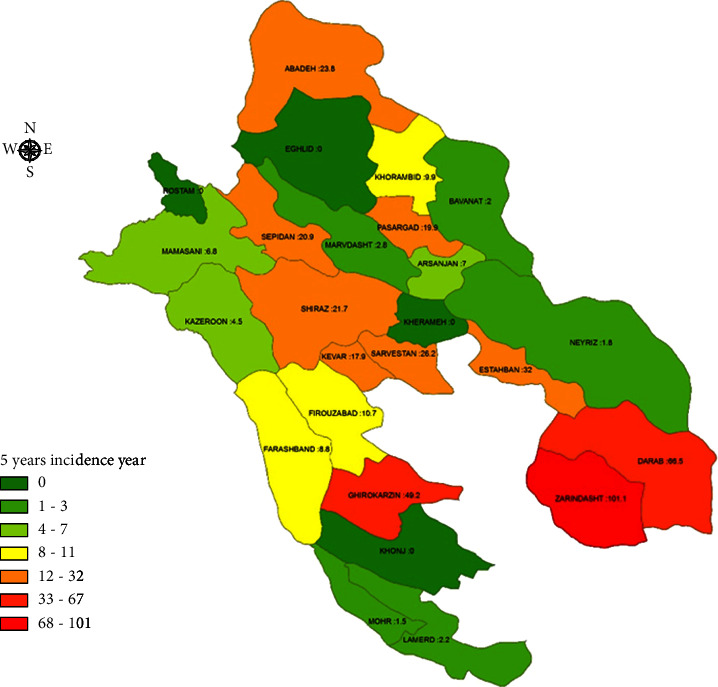
5-year incidence rate of scabies in Fars province.

**Figure 4:**
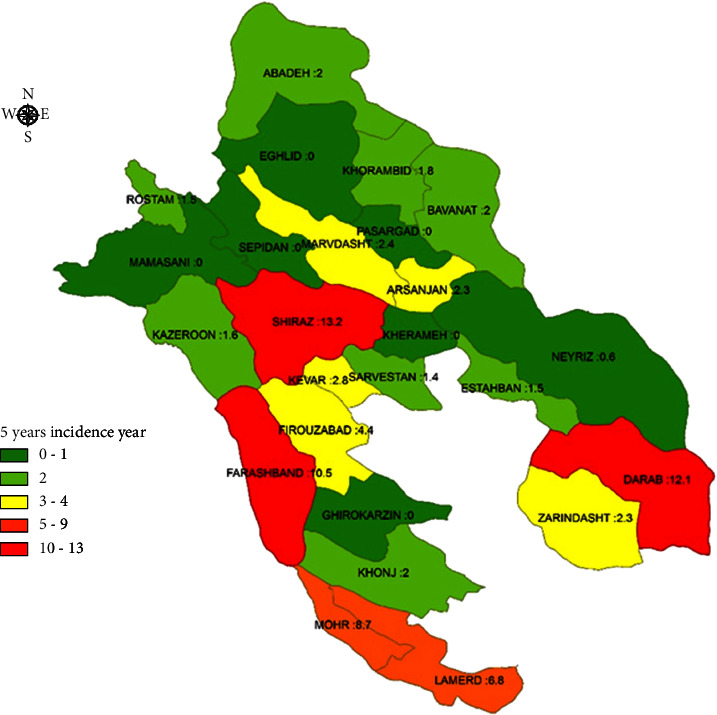
5-year incidence rate of malaria in Fars province.

Generally, the incidence of vector-borne diseases was high in the south and southwest of Fars province. The 5-year incidence of pediculosis had a maximum rate of 21,641 per 100,000 in Qir–Karzin and 20,055 per 100,000 in Zarindasht (Figure 1). Leishmaniasis was in the second stage, with an incidence of 1,915 per 100,000 in Zarindasht and 1,131 per 100,000 in Farashband (Figure 2). Zarindasht, with a scabies incidence of 101.1 per 100,000 (Figure 3), and Shiraz, with a malaria incidence of 13.2 per 100,000 (Figure 4), were the high-risk cities.

**Figure 5:**
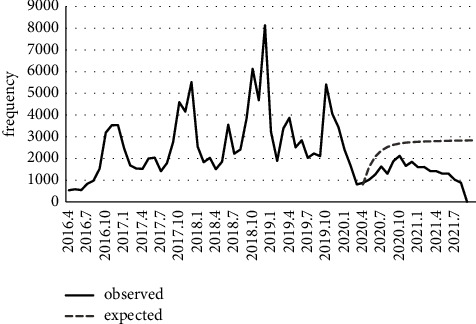
Number of reported pediculusis in Fars province and its prediction during the COVID-19 pandemic.

**Figure 6:**
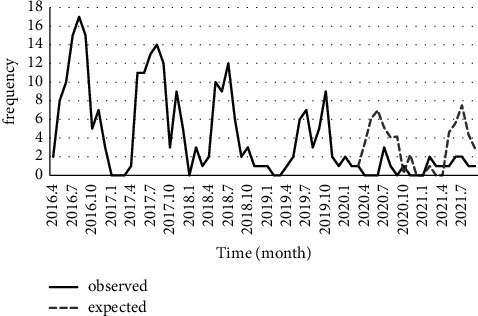
Number of reported malaria in Fars province and its prediction during the COVID-19 pandemic.

According to the time-series results, the linear trend of pediculosis was not significant (*P*=0.985). Still, there was a significant difference (*P* < 0.001) between observed and expected values before and after the COVID-19 lockdown. In other words, the ARIMA (1, 1, 1) model, which was fitted to the pediculusis data, showed that lockdown has decreased the number of reported pediculosis (Figure 5). Likewise, there was a significant decreasing trend for the data on malaria (*P* < 0.001), and the comparison of expected values of the SARIMA (0, 1, 5) (1, 1, 0) 12 model with observed values indicated that lockdown had reduced the number of malaria cases (Figure 6).

**Figure 7:**
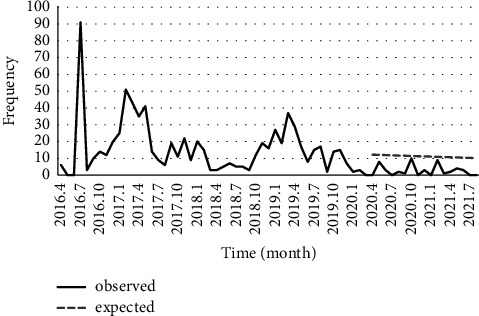
Number of reported scabies in Fars province and its prediction during the COVID-19 pandemic.

**Figure 8:**
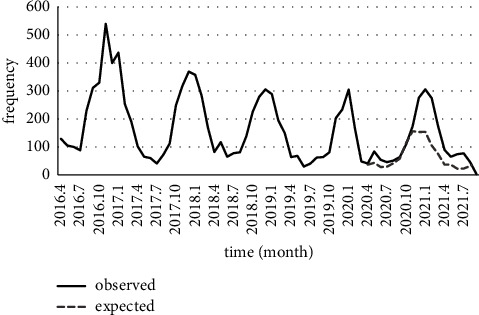
Number of reported leishmaniasis in Fars province and its prediction during the COVID-19 pandemic.

Similarly, the decreasing trend of scabies was significant (*P* < 0.001), and the fitted time-series model of MA (1) presented that COVID-19 pandemic has reduced the number of scabies cases (*P* < 0.001) (Figure 7). Also finally, although the leishmaniasis trend was decreasing (*P*=0.058), the difference between observed and expected values of the fitted model SARIMA (0, 1, 1) (1, 1, 0) 12 revealed that lockdown had increased the number of leishmaniasis cases (*P*=0.071) (Figure 8).

## 4. Discussion

VBDs are illnesses caused by pathogens that are transmitted to humans through the bites of infected vectors, such as mosquitoes, ticks, fleas, or flies. These diseases pose a significant global health threat, affecting millions of people each year. The transmission of these diseases is influenced by various factors including climate change, urbanization, and globalization, making them a growing concern in many parts of the world. Preventive measures such as vector control, vaccination, and public awareness campaigns are crucial in reducing the burden of vector-borne diseases and protecting vulnerable populations [2].

This study surveyed the change in the epidemiological profile of VBDs before and after the COVID-19 outbreak in Fars province, south of Iran. The present study was designed to evaluate the effects of COVID-19-related quarantine/isolation outcomes on the prevalence of some main ectoparasitic diseases including pediculosis and scabies in Fars province. As shown in Figure 1, Zarrin Dasht, Qirokarzin, and Mohr cities showed the highest incidence rate for pediculosis before COVID-19 outbreak among all studied areas. This phenomenon can be attributed to some epidemiological and meteorological factors, including being located in the tropical regions of the country as well as unsuitable socioeconomic and health conditions of the inhabitants of these areas.

Contrary to our prediction analysis results, after the onset of the COVID-19 pandemic, the trend of pediculosis has drastically declined in 2021 (Figure 5). Some probable factors that might affect the incidence of pediculosis are the closure of schools, limited population movements, and reduction of physical contact of people, especially among students as the high-risk group during lock down.

Pediculosis is a worldwide parasitic disease affecting school-aged children, primarily girls [15]. For the first time in the country, we investigated the effects of the COVID-19 pandemic on the infestation trend of the studied population to head lice in one of the most populated provinces of Iran.

Our statistical analysis showed that the average annual incidence rate in the two years before the COVID-19 (2018-2019) decreased from 1019.3% to 322.25% two years after the COVID-19 (2020-2021). Similarly, a significant decline in pediculosis prevalence was also reported in Argentina (Buenos Aires), from before (69.6%) to during (43.9%) COVID-19 lockdown in 1,118 children [17]. Possible reasons for this decline could be people's fear of catching the coronavirus and not going to health centers. Consequently, the number of registered cases of the disease has been reduced. Another explanation was to minimize physical contact with lockdown and implement a social distancing program [18]. Human movement restrictions of the COVID-19 lockdown significantly influenced pediculosis, fewer people available outdoors, limited human contact, and school closures and therefore diminished vector-host contact [17]. Fars province is now one of the main endemic foci of leishmaniasis in Iran due to the high spread of the disease in almost all parts of the province. The incidence of leishmaniasis has increased during the last decade [19]. Among all studied areas, Farashband and Zarrin Dasht had the highest incidence rate for leishmaniasis (Figure 2). Although the cities of Mohr and Khonj are located in the vicinity of the mentioned cities (Farashband and Zarrin Dasht), they showed a lesser number of leishmaniasis cases. Socioeconomic and educational factors differ in these two regions and can affect the epidemiology of the disease.

Leishmaniasis revealed an increasing trend during the last decade in Iran because of some reasons. In the past, when the prevalence of malaria was high in Iran, insecticides massively were applied to control different stages of anopheline mosquitoes, which consequently reduced the population of sandflies and also affected the prevalence of leishmaniasis [20]. Given that our country is in the phase of malaria elimination, malaria control operations are no longer performed, which can lead to an increase in the population of sandflies [21].

Other factor influencing the increase of the disease in recent years is the phenomenon of uncontrolled and nonstandard urban planning. Increased unprincipled urbanization is another influencing factor on transmission cycle of the disease. Almost all new settlements are being built on the margin areas of cities that are near or on the colonies of rodents. These regions usually are suitable habitats for rodents and sandflies, which can make a new focus for leishmaniasis [22].

After the coronavirus pandemic, people's desire to go to medical centers decreased sharply. Disease cases did not go to diagnostic/treatment centers, and this increased parasite reservoir hosts, especially for anthroponotic cutaneous leishmaniasis.

Iran is also one of the countries that have been severely affected by these changes, and some VBDs including leishmaniasis have also been affected by this global phenomenon [23]. Fars province is one of the main endemic foci for leishmaniasis in Iran. Three clinical forms of leishmaniasis existed in this province including zoonotic cutaneous leishmaniasis, anthroponotic cutaneous leishmaniasis, and visceral leishmaniasis. Most cases of the disease are cutaneous form [24]. People from Afghanistan and Pakistan have migrated to Iran due to facing war and economic issues, and Fars province is one of the desired destinations for refugees. These individuals can act as potential reservoirs of the disease and create the possibility of an epidemic in vulnerable parts of the province [25].

Zarrin Dasht, Qirokarzin, and Darab showed the highest 5-year incidence rate of scabies among all surveyed cities (Figure 3), which may be attributed to the health condition of the residents in these areas. Most scabies disease cases in Iran are reported from military barracks, prisons, and addiction rehabilitation centers [26]. Based on our results, the incidence rate of scabies has not significantly changed in the observed and predicted value before and after occurrence of COVID-19 pandemic (Figure 7). Scabies is an ectoparasitic disease often reported in places which have not become less crowded since the outbreak of COVID-19. Social distancing and reducing the number of people in such sites are impractical.

Because of various equipped medical centers, Shiraz is an ideal destination for all patients in south Iran. Accordingly, most malaria patients refer to Shiraz for treatment, so most of the reported malaria cases in Shiraz are imported (Figure 4). The results of this study depicted that malaria in different regions of Fars province has a significantly lower prevalence than years before the COVID-19 pandemic (Figure 6). Malaria and COVID-19 can have similar clinical manifestations, including fever, sweats and chills, headaches, loss of appetite, muscle pains, sore throat, cough, and difficulty breathing. These common symptoms may lead to misdiagnosis of malaria and COVID-19, especially when the doctors only pay attention to disease symptoms [27]. In Taiwan, a study by Lai et al. investigated the effect of the coronavirus disease epidemic on notifiable infectious diseases. Seven routine VBDs showed an overall reduction of 557 (−54.8%) cases from 2019 to 2020. Similar trends were observed for the changes in incidence, which were 2.4 (−55.0%) per 100,000 people for vector-borne diseases [28].

Since the tropical regions of Iran are considered recreational and touristic areas, during COVID-19 outbreaks, some reasons such as the restrictions on travelers' movement and the implementation of general quarantine, caused people to receive fewer bites from mosquitoes and other blood-sucking arthropods in these areas.

Another research considered the effect of coronavirus disinfectants “sodium hypochlorite” on mosquito populations in southwestern Iran (Abadan and Khorramshahr city). They showed that excessive use of disinfectants releases large amounts of chlorine gas into the environment, which may disrupt the natural balance of ecosystems. They indicated that the decrease in the mosquito population is related to the entry of sodium hypochlorite into the environment [29].

In addition, quarantine, movement restrictions, obligatory isolation, and reduction in night movement reduced the contact between mosquitoes and the residents of the area. It may be another possible reason for the decrease in malaria cases in Fars province [6].

## 5. Conclusion

The pandemic of COVID-19 caused tremendous changes in human lifestyle and the epidemiological pattern of many diseases. Some of the consequences of the COVID-19 can occur in the long term and affect populations after many years. Since the transmission of vector-borne diseases is very complex and many parameters affect them, the epidemiological profile of VBDs should also be discussed and investigated in the post-COVID-19 era.

## Figures and Tables

**Table 1:** Annual incidence rate (no. of cases per 100,000) of main vector-borne disease.

	Scabies	Leishmaniasis	Pediculusis	Malaria
2016	7.3	82.9	565.6	2.2
2017	5.4	58.6	864.4	2.2
2018	4.2	53.5	1146	1.3
2019	3.4	36.3	892.6	1.1
2020	1	44	486.7	0.2
2021	0.2	9.4	157.8	0.2

## Data Availability

The data that support the findings of this study are available from the corresponding author upon reasonable request.
